# A lack of drebrin causes olfactory impairment

**DOI:** 10.1002/brb3.3354

**Published:** 2023-12-31

**Authors:** Yuki Kajita, Nobuhiko Kojima, Tomoaki Shirao

**Affiliations:** ^1^ Department of Neurobiology & Behavior Gunma University Graduate School of Medicine Maebashi Gunma Japan; ^2^ Faculty of Life Sciences Toyo University Ora‐gun Gunma Japan; ^3^ AlzMed, Inc., UT South Building, Entrepreneurs Laboratory Bunkyo‐ku Tokyo Japan

**Keywords:** Alzheimer's disease, behavior test, knockout mouse, olfaction

## Abstract

**Introduction:**

Olfactory deficit often occurs during the prodromal stage of Alzheimer's disease (AD). Although olfactory deficit is a useful measure for screening AD‐related amnestic disorder, little is known about the cause of this deficit. Human and animal studies indicate that loss of the actin binding protein, drebrin, is closely related to cognitive dysfunction in AD. We hypothesized that the olfactory deficit in AD is caused by the loss of drebrin from the spine.

**Methods:**

To verify this hypothesis, we performed the buried food test in two types of drebrin knockout mice, such as drebrin‐double (E and A) knockout (DXKO) mice, and drebrin A‐specific knockout (DAKO) mice.

**Results:**

The DXKO mice spent a significantly longer time to find food compared with the wild‐type (WT) littermates. In contrast, the DAKO mice, in which drebrin E rather than drebrin A is expressed in the postsynaptic sites of mature neurons, spent an equivalent time trying to find food compared to that of the WT. The DXKO mice showed comparable food motivation and sensory functions other than olfaction, including visual and auditory functions.

**Conclusion:**

These results indicate that drebrin is necessary for normal olfactory function. Further study is needed to determine whether it is necessary for normal olfaction to express drebrin E during the developmental stage or to have drebrin (whether E or A) present after maturation.

## Graphical abstract

Drebrin double knockout impairs olfaction. Drebrin A‐specific knockout does not impair olfaction. Senses other than olfaction are normal in drebrin double knockout mice.

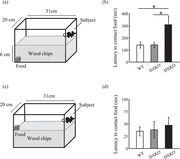

## 1 INTRODUCTION

Olfaction is extremely important for survival in most vertebrates, and olfactory dysfunction is closely related to several cognitive disorders. Olfactory deficit often occurs during the prodromal stage of Alzheimer's disease (AD), and olfactory deficit is a useful measure for screening AD‐related amnestic disorder. Although synaptic functions are crucial for processing odor signals, little is known about the cause of the olfactory deficit in AD.

Drebrin forms a stable actin filament with a long cross over, which accumulates in postsynaptic sites and is involved in synaptic function (Shirao et al., 2017). Previous studies have shown that expression of the drebrin protein and mRNA decreases in the AD human hippocampus, frontal cortex, and temporal cortex, although the levels of presynaptic proteins, such as synapsin I and synaptophysin, remain unchanged (Harigaya et al., 1996; Julien et al., 2008; Shim & Lubec, 2002; Shirao et al., 2017). Interestingly, drebrin expression also decreases in patients with mild cognitive impairment (MCI), a putative prodromal stage of AD, in temporal cortex and the hippocampus (Counts et al., 2012, 2006). This result indicates that reduced drebrin is a hallmark of AD brains from the early pathological stage (Ishizuka & Hanamura, 2017).

Because drebrin has a crucial role in synaptic function (Koganezawa et al., 2017; Sekino et al., 2017), drebrin knockout causes cognitive disorder in animal experiments (Jung et al., 2015; Kojima et al., 2016). However, olfactory deficits have never been examined. Olfactory deficits are evident in patients with MCI and may predict the progression of MCI to AD (Attems et al., 2014; Fatuzzo et al., 2023). Thus, olfactory deficit is used to stratify the risk of conversion from MCI to AD (Woodward et al., 2017). Although the mechanism of olfactory impairment in the early stage of AD has not been clarified, we hypothesized that synaptic dysfunction caused by the loss of drebrin may be involved.

In the present study, to verify this hypothesis, we examined the olfactory function of drebrin knockout mice and found that drebrin‐double (embryonic isoform: E and adult isoform: A) knockout (DXKO) but not drebrin A‐specific knockout (DAKO) caused olfactory impairment.

## 2 MATERIALS AND METHODS

### 2.1 Animals

DXKO (Kajita et al., 2017) and DAKO mice (Kojima et al., 2010) were generated previously and passaged in a C57BL/6N genetic background. Wild‐type (WT) littermates were used as the control. Male 5‐month‐old DXKO, DAKO, and WT mice (weight 25–35 g) were used in all experiments. These mice were housed in groups in a temperature‐ and humidity‐controlled room under a 12 h light/dark cycle with food and water available ad libitum. Efforts were made to minimize the number of animals used, as well as their pain and discomfort. All animal treatments were performed following the regulations outlined by Japanese law and the National Institutes of Health guidelines and were approved by the Animal Care and Experimentation Committee of Gunma University, Showa Campus (Maebashi, Japan).

### 2.2 Behavioral testing

All behavioral tests were performed between 1:00 p.m. and 5:00 p.m. All equipment except for the buried food test and taste preference test used in the behavioral experiments was manufactured by O'Hara and Co. The apparatus were wiped with alcohol before each test and the standing position of the experimenter was fixed for all experiments. Behavioral tests were described later, in order of trials. The details of the procedures for the open field and light/dark transition tests followed Kojima et al. (2010). The method of the acoustic startle reflex test followed Fujihara et al. (2015).

#### 2.2.1 Buried food test

Olfaction was evaluated using the buried food test slightly modified from Yang and Crawley (2009). Mice (WT, *n* = 34 mice; DAKO, *n* = 19 mice; DXKO, *n* = 20 mice) were food‐restricted to lose 10% of their body weight. A 3‐g piece of palatable food (CalorieMate cheese flavor, Otsuka Pharmaceutical Co., Ltd.) was buried in the corner of nesting wood chips (6 cm in depth) in a cage (plastic translucent walls and floor, 20 × 31 cm^2^ (Harigaya et al., 1996)), and the mice were placed on the diagonally opposite corner. The latency for the mice to find the buried food was measured manually. The motivation to search for food was compared with the other tests. This test was conducted in the same way as the buried food test, except that food was not buried but was just placed on top of the wood chips to be visually recognized by the mice.

#### 2.2.2 Taste preference test

Gustation was evaluated by the taste preference test. The sense of taste is measured as a selective test (Riley & Freeman, 2004). Mice generally prefer intensely flavored food to normal pellet food. The mice (WT, *n* = 20 mice; DXKO, *n* = 20 mice) were food restricted to lose 10% of their body weight. Before the test, the mice were fed a palatable food (CalorieMate cheese flavor, Otsuka Pharmaceutical Co., Ltd.) and a normal pellet food to learn the taste of each food. The piece of palatable food was placed 30 cm away from the mouse and a normal food pellet was placed between the palatable food and the mouse. We observed which food was selected first by the mouse.

#### 2.2.3 Open field test

Spontaneous activity, exploratory behavior, and the emotional response to a novel environment were measured in the open field test (Plexiglas white walls and floor, 50 × 50 cm^2^ (Harigaya et al., 1996); 100 lx). The mice (WT, *n* = 11 mice; DXKO, *n* = 10 mice) were placed in the corner and their movements were tracked for 15 min using a digital camera equipped with tracking software. Rearing was detected by an infrared photo‐beam detection system. The distance traveled, number of rearings, and time spent in the central square (25 × 25 cm^2^ (Harigaya et al., 1996)) of the field were measured.

#### 2.2.4 Light/dark transition test

Visual perception was measured by the light/dark transition test. A dark compartment (20 × 20 cm^2^ [Harigaya et al., 1996]; 5 lx) was connected to a brightly lit compartment (20 × 20 cm^2^ (Harigaya et al., 1996); 200 lx) via one aperture (3 cm wide × 4 cm height). The mice (WT, *n* = 11 mice; DXKO, *n* = 10 mice) were placed in the dark compartment and the number of transitions between compartments and the total time in the brightly lit compartment was recorded for 10 min using a digital camera equipped with tracking software.

#### 2.2.5 Rotarod test

Motor coordination was measured by the rotarod test. The mice (WT, *n* = 11 mice; DXKO, *n* = 10 mice) were placed on the rotating rotarod apparatus. The time that the animal could hold itself on the rod (*φ* 32 mm) was recorded using an infrared photo‐beam detection system for up to 400 s during which the rotation speed gradually increased from 3 to 40 rpm. The test was conducted four times, and each trial interval was 20 min. The total time of the four trials was used as the score.

#### 2.2.6 Thermal gradient test

The response to pain was measured by the thermal gradient test. The paws of mice are very sensitive to heat at temperatures that are not damaging to the skin. A gradient hot plate apparatus was used, which consisted of 17 metal plates for which the temperature ranged from 15 to 55°C. The mice (WT, *n* = 11 mice; DXKO, *n* = 10 mice) were placed on the 15°C plate and the time remaining on each plate was recorded for 2 h using a digital camera equipped with tracking software. Data from the last 30 min were used to avoid exploratory behavior.

#### 2.2.7 Acoustic startle reflex test

Auditory acuity was measured by the acoustic startle reflex test (WT, *n* = 10 mice; DXKO, *n* = 10 mice). The test session began by placing a mouse undisturbed in a Plexiglas cylinder for 5 min. The background noise level in the chamber was 10 dB. The startle reflex was recorded for 140 ms (response measured every 1 ms). Five bursts of noise (70, 75, 80, 85, and 90 dB) were delivered repeatedly in quasi‐random order at random inter‐trial intervals (10–20 s). High‐intensity sound (>120 dB) is likely to cause the startle reflex through a tactile sense rather than auditory perception; therefore, only comparable mild sounds were selected. A piezoelectric accelerometer transduced the displacement of the test cylinders in response to animal movement.

### 2.3 Statistical analysis

We used SPSS software (IBM Corp. Released 2012. IBM SPSS Statistics for Windows, Version 21.0) for the statistical analysis and performed Welch's *t*‐test and one‐ or two‐way analysis of variance followed by post hoc Scheffe and Tukey HSD tests. Numerical analyses were also performed with SPSS software. Values are presented as mean ± SEM. *p*‐Values < .05 were considered significant.

## 3 RESULTS

### 3.1 DXKO mice exhibit olfactory defects

**FIGURE 1:**
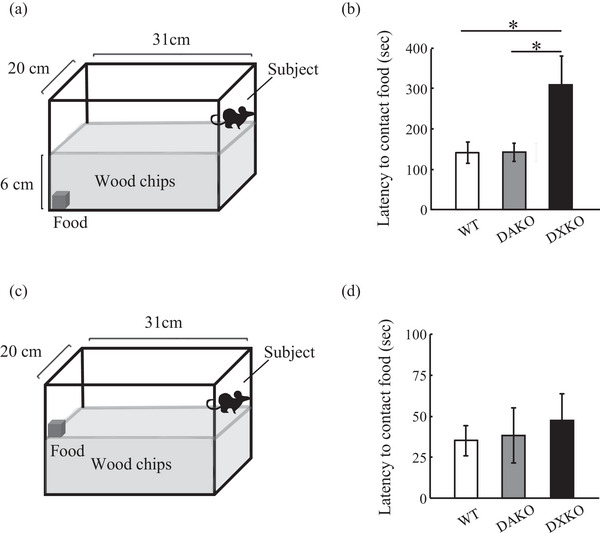
Drebrin‐double (E and A) knockout (DXKO) mice exhibit abnormal behavior on the buried food test: (a) The buried food test measures olfactory acuity; (b) the latency for DXKO (*n* = 20 mice), drebrin A‐specific knockout (DAKO) (*n* = 19 mice), and wild‐type (WT) (*n* = 34 mice) mice to find food; (c) the test measures the motivation of the mice for food; and (d) the latency for DXKO (*n* = 20 mice), DAKO (*n* = 19 mice) and WT (*n* = 34 mice) mice to contact the food. Error bars indicate SEM. One‐way ANOVA, post hoc Scheffe's test was used in (b) and (d). *p < .05

The buried food test apparatus used in this experiment is depicted in Figure 1a. We performed the buried food test using drebrin knockout mice. All the genotypes of mice found the buried food, but the DXKO mice spent twice as long looking compared to the WT mice and the DAKO mice (Figure 1b; WT, 141.2 ± 26.6 s; DAKO, 142.3 ± 22.4 s; DXKO, 307.5 ± 71.4 s; WT to DAKO, *p* = 1.0; WT to DXKO, *p* = .018; DAKO to DXKO, *p* = .047; WT, *n* = 34; DAKO, *n* = 19; DXKO, *n* = 20).

The motivation to find food is an important factor in the buried food test. In this test, food was placed on the surface of the wood chips instead of buried, but other conditions were performed in the same way as the buried food test (Figure 1c). The latency to contact the food did not differ between the DXKO mice and WT and DAKO mice (Figure 1d; WT, 35.2 ± 9.1 s; DAKO, 38.1 ± 16.8 s; DXKO, 47.1 ± 16.0 s; WT to DAKO, *p* = .98; WT to DXKO, *p* = .79; DAKO to DXKO, *p* = .91; WT, *n* = 34; DAKO, *n* = 19; DXKO, *n* = 20). These data suggest that the motivation to find the food did not differ among the animal groups and was unlikely to be a causal factor for the impairment on the buried food test in the DXKO mice.

Additionally, we tested curiosity for a palatable food on the taste preference test, because the DXKO mice may not prefer the food (CalorieMate cheese flavor). In this test, all DXKO mice chose the palatable food rather than the normal pellet food, similar to the WT mice (WT, 20/20 mice; DXKO, 20/20 mice).

### 3.2 DXKO mice exhibit normal locomotor activity, anxiety level, and motor function

**FIGURE 2:**
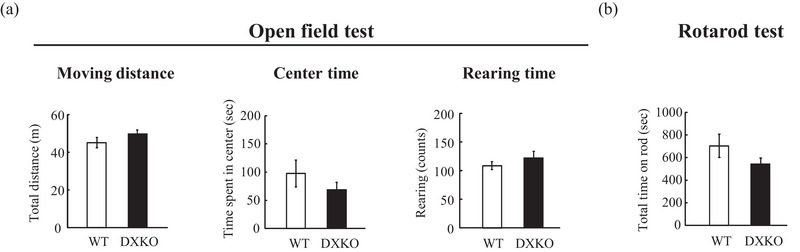
Drebrin‐double (E and A) knockout (DXKO) mice exhibit normal behavior on the open field and rotarod tests: (a) the open field test measures locomotor activity and anxiety level in the mice. Total distance moved (left), time spent in the center area (middle), and rearing time (right) were measured, respectively (wild‐type [WT], *n* = 11 mice; DXKO, *n* = 10 mice); (b) the rotarod test measures motor function. The time spent riding the rod was measured (WT, *n* = 11 mice; DXKO, *n* = 10 mice). Error bars indicate SEM. Welch's *t*‐test was used in (a) and (b).

Next, we excluded other causal factors for the impairment on the buried food test, for example, locomotion activity, anxiety level, and motor function. We examined these functions using the open field and rotarod tests. In the open field test, we measured the total distance traveled as locomotor activity; the time spent in the center field and the rearing time represented the anxiety level. No significant differences in distance traveled, center time or rearing time was observed between the groups (Figure 2a; moving distance; WT, 45.0 ± 2.7 m; DXKO, 49.7 ± 20.1 m; *p* = .18, center time; WT, 97.5 ± 23.9 s; DXKO, 69.1 ± 12.8 s; *p* = .32, rearing time; WT, 215.0 ± 23.9 times; DXKO, 255.1 ± 12.8 times; *p* = .50; WT, *n* = 11; DXKO, *n* = 10).

In the rotarod test, we measured the time spent running on a moving wheel as the motor function. No significant difference in total running time was detected between DXKO and WT mice (Figure 2b; WT, 703.9 ± 103.9 s; DXKO, 541.8 ± 53.4 s; *p* = .19; WT, *n* = 11; DXKO, *n* = 10). These results suggest that locomotor activity, anxiety level, and motor function were not causal factors for the impairment on the buried food test in DXKO mice.

### 3.3 DXKO mice have normal visual, nociceptive, and auditory senses

**FIGURE 3:**
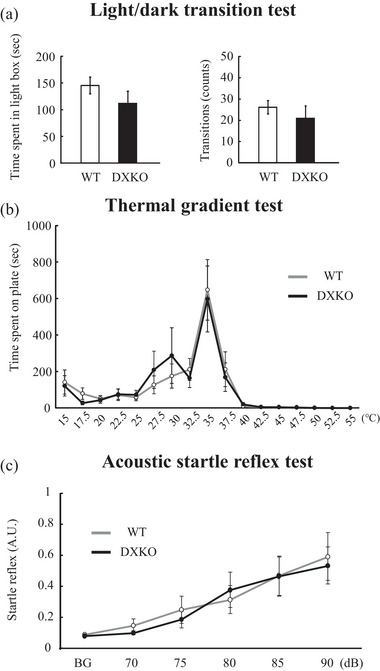
Drebrin‐double (E and A) knockout (DXKO) mice exhibit normal behavior on the light/dark transition, thermal gradient, and acoustic startle reflex tests. (a) The light/dark transition test measures visual acuity. The time spent in the lightbox (left) and the number of transitions between the boxes (light) were measured (wild‐type [WT], *n* = 11 mice; DXKO, *n* = 10 mice). (b) The thermal gradient test measures pain sensitivity. The time spent on each of the 17 plates was measured (WT, *n* = 11 mice; DXKO, *n* = 10 mice). (c) The acoustic startle reflex test measures auditory acuity. All sound‐induced (70–90 dB) startle reflex levels were measured (WT, *n* = 10 mice; DXKO, *n* = 10 mice). Error bars indicate SEM. Welch's *t*‐test was used in A. Two‐way ANOVA, post hoc Tukey HSD test was used in (b) and (c).

We tested the visual, nociceptive, and auditory senses to determine whether DXKO mice specifically show olfactory impairment or ubiquitously show other sensory impairments. We measured the visual sense (discrimination ability of light and darkness) in the light/dark transition test. Neither the time spent in the light box nor the number of transitions between the two boxes changed significantly (Figure 3a; time spent in light box; WT, 145.2 ± 15.6 s; DXKO, 112.1 ± 22.4 s; *p* = .21, number of transitions; WT, 26.0 ± 3.0 times; DXKO, 21.0 ± 5.7 times; *p* = .40; WT, *n* = 11; DXKO, *n* = 10). Because the open field test in Figure 2 shows that the anxiety level of the DXKO mice was similar to that of WT mice, the anxiety level did not affect the light/dark transition test.

Nociception was measured by the thermal gradient test, which reflects thermal nociception. When the mice were placed on the plate, they moved through the plates and finally stopped on the plate at the optimal temperature. The DXKO and WT mice spent more than one‐third of their time on the center plate which was 35°C, and no significant difference was detected in the time spent on the other 17 plates (Figure 3b; *p* = 1.00; WT, *n* = 11; DXKO, *n* = 10).

Auditory acuity was measured by the acoustic startle reflex test. The amplitudes of the reflex to the bursts of the noise were comparable between DXKO and WT mice (Figure 3c; *p* = .77; WT, *n* = 10; DXKO, *n* = 10).

## 4 DISCUSSION

### 4.1 Difference in the olfactory phenotypes between DXKO and DAKO mice

#### 4.1.1 Drebrin E compensates for a lack of drebrin A

Drebrin E is the embryonic isoform in the developing brain that is replaced with drebrin A as the neuron‐specific isoform in the adult brain. In this study, we demonstrated that olfaction was not impaired in DAKO but was impaired in DXKO mice. This result suggests that drebrin E compensates for the role of drebrin A in adult olfactory function.

Drebrin E shares an amino acid sequence with drebrin A except an insertion of 46 amino acids named Ins2 (Kojima et al., 1993). Common N‐terminal region (aa 1–300) stabilizes F‐actin (Mikati et al., 2013), and drebrin E localizes in dendritic spines where drebrin A is lacking (Kojima et al., 2010). Another knockout mouse line lacking both drebrin E and A has been reported to cause abnormalities in the morphology of the dendritic spine and memory‐related synaptic plasticity in adult (11‐week old) hippocampus (Jung et al., 2015), but the adolescent (8–9‐week old) and young adult (16–20‐week old) our DAKO mice lacking only drebrin A exhibited normal hippocampal long‐term potentiation and hippocampal‐dependent fear learning (Kojima et al., 2010, 2016). These previous reports support our hypothesis that drebrin E compensates for some parts of the role of drebrin A in synaptic regulation.

However, the compensation of drebrin E for drebrin A is insufficient in juvenile and aging brains. In juvenile (2‐week old) DAKO brain, metabotropic glutamate receptor‐dependent long‐term depression is enhanced in the hippocampus compared with that in WT mice (Yasuda et al., 2018). In contrast, in aging (28–42‐week old) DAKO brain, the morphology of hippocampal dendritic spines and memory‐related synaptic plasticity are impaired (Kojima et al., 2016). These studies suggest that the role of drebrin E at postsynaptic sites is insufficient for age‐dependent synaptic plasticity. The failure of synaptic remodeling due to the loss of drebrin may reflect the symptoms of AD with aging.

#### 4.1.2 Adult neurogenesis is disturbed in DXKO mice

Olfactory inhibitory interneurons are a restricted neuron that is replaced by newly generated neurons in the adult brain (Lois & Alvarez‐Buylla, 1993). We have previously shown that adult neurogenesis is partly disturbed in DXKO mice, but not in DAKO mice (Kajita et al., 2017), possibly due to dysregulation of migration of newly generated neurons (Sonego et al., 2015; Trivedi et al., 2017). Daily realignment of neural circuits in the olfactory bulb (OB) by newly generated neurons is essential to maintain normal olfactory function. Deficits in cell migration induced by neural cell adhesion molecule‐deficient mice result in impairments in the olfactory discrimination task (Gheusi et al., 2000). However, the loss of the neural cell adhesion molecule affects neurogenesis and the formation of the neural circuit. Neurogenesis decreases in response to gamma‐ray irradiation, which affects olfactory‐dependent learning, but not the olfactory discrimination task (Lazarini et al., 2009). Further, in recent experiments, genetic ablation of newly generated neurons in the OB affects predator avoidance and sexual behaviors, but not the response to odors (Sakamoto et al., 2011). In addition, the decrease in neurogenesis from aging does not impair the ability to discriminate different odors, although it causes difficulties with precise discrimination between similar odors (Enwere et al., 2004). These studies indicate that the impaired olfactory acuity in DXKO mice may be independent of the aberrant incorporation of newly generated neurons.

### 4.2 Possible causes of olfactory dysfunction in DXKO mice

Drebrin A is widely distributed in the adult forebrain, but no other sensory defects are found in DXKO mice except olfaction. Unlike the other senses, the information of smell is not directly transmitted to the thalamus but is transmitted to the olfactory cortices, including the olfactory tubercle, piriform cortex, amygdala, and entorhinal cortex (EC), suggesting that neural regulation in these regions plays the role of gatekeeper in the olfactory system. In particular, the EC often exhibits histological changes during the early stages of AD (Khan et al., 2014), and impaired neuronal activity in the EC precedes neurodegeneration, indicating that symptoms during the early stage of AD are likely to be caused by dysfunctions of activity rather than by cell death (Igarashi, 2023). In addition, the loss of drebrin causes a pathological neuronal firing pattern in the EC (Klemz et al., 2021). These studies suggest that the cause of the olfactory impairment in DXKO mice is abnormal neuronal activity in the olfactory cortices, particularly the EC.

Odors are detected by odorant receptors in the olfactory epithelium, which is necessary for olfaction (Slotnick et al., 2007). Drebrin E is expressed in olfactory epithelial cells where it regulates the developmental transition from basal progenitor cells to olfactory sensory neurons (McIntyre et al., 2010). A second possible cause of the olfactory impairment in DXKO mice is developmental abnormality of olfactory sensory neurons in the olfactory epithelium.

Olfactory sensory neurons in the olfactory epithelium transmit signals to mitral cells in the OB. The mitral and tufted cells are the primary output neurons and the central relay in the olfactory system of the brain. Thus, a final possible cause is abnormal signal transduction from output neurons in the OB to the olfactory cortices. Further studies are necessary to compare neural activity in the whole olfactory pathway between DXKO and WT mice to determine which possibility is likely to explain the olfactory disability in DXKO mice.

The olfactory dysfunction observed in DXKO mice suggests that a lack of drebrin results in abnormalities in any of the neural circuits associated with the olfactory system. However, the lack of drebrin does not immediately lead to neuronal degeneration, as drebrin knockout mice do not show neuronal degeneration as observed in AD (Kajita et al., 2017). In addition, it is unclear whether it is necessary for normal olfaction to express drebrin E at the developmental stage or whether drebrin (whether E or A) is present in the spine after maturation. Therefore, detailed studies, including cellular and neural circuit levels, are needed to determine whether DXKO brains may aid in elucidating the mechanisms of olfactory dysfunction during the progression of neurodegenerative diseases, such as AD.

## 5 CONCLUSION

We hypothesized that a lack of synaptic drebrin may contribute to cognitive decline in AD, because drebrin reduction is observed in parallel with cognitive decline in MCI and the preclinical stages of AD. Several behavioral studies have shown that a lack of drebrin causes cognitive impairment. However, no study has reported on olfactory impairment in drebrin KO animals, which is a useful measure for diagnosing AD. Therefore, we performed several behavioral tests using two types of genetically modified mice and found that DXKO, but not DAKO mice, had impaired olfaction. These results suggest that the expression of drebrin E or both isoforms of drebrin (drebrin E and A) is necessary to maintain normal olfactory function.

## AUTHOR CONTRIBUTIONS

Conceptualization, YK and NK; methodology, YK and NK; validation, YK and NK; formal analysis, YK; investigation, YK; resources, NK and TS; data curation, YK; writing—original draft preparation, YK, NK, and TS; writing—review and editing, YK, NK, and TS; visualization, YK; supervision, NK and TS; project administration, NK and TS; funding acquisition, NK and TS. All authors have read and agreed to the published version of the manuscript.

### PEER REVIEW

The peer review history for this article is available at https://publons.com/publon/10.1002/brb3.3354.

## Data Availability

The raw data supporting the conclusions of this article will be made available by the authors, without undue reservation.
